# Study on Differential Protein Expression in Natural Selenium-Enriched and Non-Selenium-Enriched Rice Based on iTRAQ Quantitative Proteomics

**DOI:** 10.3390/biom9040130

**Published:** 2019-03-30

**Authors:** Rui Zeng, Muhammad Umer Farooq, Li Wang, Yang Su, Tengda Zheng, Xiaoying Ye, Xiaomei Jia, Jianqing Zhu

**Affiliations:** 1 Demonstration Base for International Science & Technology Cooperation of Sichuan Province, Rice Research Institute, Sichuan Agricultural University, Chengdu 611130, Sichuan, China; zengrui829@163.com (R.Z.); umerpbguaf@gmail.com (M.U.F.); imsuxiaomeng@163.com (Y.S.); 13089068357@163.com (T.Z.); yeyixuana@163.com (X.Y.); jessicamei2372@sina.com (X.J.); 2 Dujiangyan Agricultural and Rural Bureau, Dujiangyan 611830, Sichuan, China; 3 Meishan Vocational & Technical College, Meishan 62000, Sichuan, China; wli_1981@163.com

**Keywords:** rice, natural selenium-enriched, non-selenium-enriched, proteomics, iTRAQ

## Abstract

This work was designated to scrutinize the protein differential expression in natural selenium-enriched and non-selenium-enriched rice using the Isobaric-tags for relative and absolute quantification (iTRAQ) proteomics approach. The extracted proteins were subjected to enzyme digestion, desalting, and identified by iTRAQ coupled with liquid chromatography-tandem mass spectrometry (LC-MS/MS) technology. High pH C18 separation analysis was performed, and the data were then analyzed by Protein Pilot^TM^ (V4.5) search engine. Protein differential expression was searched out by comparing relatively quantified proteins. The analysis was conducted using gene ontology (GO), cluster of orthologous groups of proteins (COG) and Kyoto encyclopedia of genes and genomes (KEGG) metabolic pathways. A total of 3235 proteins were detected and 3161 proteins were quantified, of which 401 were differential proteins. 208 down-regulated and 193 up-regulated proteins were unveiled. 77 targeted significant differentially expressed proteins were screened out for further analysis, and were classified into 10 categories: oxidoreductases, transferases, isomerases, heat shock proteins, lyases, hydrolases, ligases, synthetases, tubulin, and actin. The results indicated that the anti-stress, anti-oxidation, active oxygen metabolism, carbohydrate and amino acid metabolism of natural selenium-enriched rice was higher than that of non-selenium rice. The activation of the starch synthesis pathway was found to be bounteous in non-selenium-enriched rice. Cysteine synthase (CYS) and methyltransferase (metE) might be the two key proteins that cause amino acid differences. OsAPx02, CatC, riPHGPX, HSP70 and HSP90 might be the key enzymes regulating antioxidant and anti-stress effect differences in two types of rice. This study provides basic information about deviations in protein mechanism and secondary metabolites in selenium-enriched and non-selenium-enriched rice.

## 1. Introduction

Selenium (Se) has many effects on the growth and development of plants. It regulates photosynthesis, respiration, enhances stress resistance, and attenuates the damage of free radicals, while also mitigating the toxic effects of heavy metals [1,2,3]. The absorption and accretion of Se by various plants varies greatly. Cruciferous plants have a relatively strong ability to accumulate Se, followed by legumes and then cereals [4]. The Se enrichment of crop plant mainly focuses on two aspects: one is to improve the Se content in plants during physiological cultivation by fertilization; the other is to acclimatize and breed the Se-enriched parents. Foliar Se application, soil treatment and seed dressing methods can be deployed to enrich plants with Se. However, each method has its own restrictions. Soil is the main source of nutrition in plants, as far as Se application in soil is concerned. A number of methods are implemented to enrich plants with Se (i.e., soil Se treatment, seed dressing and foliar Se fertilization). The effectiveness in Se absorption by rice can be distorted by many factors, such as Se content in the soil, Se forms, alkalinity/acidity of soil, and metal-ion interaction [5,6,7]. However, the success rate by the seed dressing Se application method is limited, and dosage is difficult to control. Moreover, this method found a significant difference in Se distribution in the various organs of rice plants, and relatively low Se content in rice grains [8]. The fertilizer efficiency by foliar spray method is short—it cannot be applied in rainy or windy conditions. It also results in easy leaching by rainwater, leading to contamination of the surrounding environment. Moreover, the amount of foliar spray is difficult to control, and it is quickly lost from the surface of hydrophobic leaves. As it is difficult to transfer the sprayed element from the absorption site of the leaf to other parts of the plant, the spraying effects are rather poor. In addition, over-spraying of Se may result in poisoning of the plant. These are the obstacles that rice breeders face when they attempt Se-enrichment of their plants. About half of the world’s population consumes rice (*Oryza sativa* L.) as a staple food. Thus, its biofortification by means of breeding is a long-lasting and comparatively riskless process. Rice genome is sequenced and studied more than other crops [9]. In addition, the molecular mechanism of Se-enrichment in rice is unclear. However, there are limited studies on differential proteinic expression in crop plants with response to Se. Therefore, the need of the hour is to assess the natural proteinic response of rice towards Se-enrichment and to compare it with non-Se food.

Isobaric-tags for relative and absolute quantification (iTRAQ) technology is an isobaric labelling method used in quantitative proteomics by tandem mass spectrometry to determine the amount of proteins from different sources in a single experiment. It uses stable isotope labelled molecules to perform accurate qualitative identification and quantitative analysis of sample proteins or peptide fragments. It can make an absolute or relative content comparison of proteins from different samples to identify differential proteins and functions [10]. The iTRAQ method has good quantification and high reproducibility and can scientifically reveal the dynamic changes of intracellular proteins in different physiological states. At present, studies on rice proteomics are rapidly emerging. The expression pattern of various tissues, organs, and sub-cells have been studied to identify the specificity of each tissue and organ in various growth and developmental stages of rice [11,12,13,14,15]. Mutant and hormone-induced proteomic studies have been conducted to unveil relevant genetic information and hormone signalling patterns [16,17]. The adverse effects of stress on rice growth and rice recovery have also been identified over time. The differentially expressed proteins have already been quantified and compared in response to environmental stress and adaptation processes [18,19,20]. However, limited studies can be found on the quality and nutritional aspects of rice.

In this experiment, an iTRAQ proteomic approach was used to investigate the differential proteinic expression in natural Se-enriched and non-Se-enriched rice at the proteome and metabolome levels. The Gene Ontology (GO) and Kyoto Encyclopedia of Genes and Genomes (KEGG) metabolic pathways were used to analyze the differential protein profiles and signalling pathways related to rice biology. The functional annotation and characterization of these proteins will provide the basic information and be helpful to scrutinize Se responsive mechanisms. The signal pathways identified by the bioinformatic analysis and their verification will provide a basis for further research on the anti-oxidation and anti-ageing mechanisms of Se-enriched rice.

## 2. Materials and Methods

### 2.1. Germplasm Collection

Two rice cultivars; Z3057B (Se-enriched) labelled as S3057, and Chenghui 727 (non-Se-enriched) labelled as S727 were provided by the Demonstration Base for International Science and Technology Cooperation, Rice Research Institute of Sichuan Agricultural University (Chendu, Sichuan, China). Using heterosis [21], the material was cross-bred over years to have a bioaccumulation effect on Se. The material (S3057) was tested by the Rice Testing Center of the Ministry of Agriculture (Chendu, Sichuan, China) and 0.046 mg kg^−1^ of Se content was found to have accumulated in polished rice, which meets the national standard of rich-Se paddy, that is, 0.04–0.30 mg kg^−1^ (GB/T 22499-2008). 

### 2.2. Protein Extraction

Total protein was extracted from rice grain samples. 5 g of grains powder was dissolved using 200 μL of TEAB dissolution buffer. The dissolution process was boosted up by 15 min ultrasonication (WD-9415B, LiuYi Co., Ltd., Beijing, China). The dissolved mixture was then centrifuged (12,000 r/min, 20 min, at 4 °C) and the supernatant was subsided by adding 4-volume dithiothreitol (DTT, 10 mM) in cold acetone for 2 h. The suspension was centrifuged (12,000 r/min, 20 min, at 4 °C). The precipitate was collected and mixed with cold acetone (800 μL, at 56 °C) to break the proteins’ disulfide bond. The mixture was then centrifuged again (12,000 r/min, 20 min, at 4 °C) and the pellet dried. Finally, the dried pellet was collected and dissolved in dissolution buffer (100 μL Triethylamine borane) and stored at −80 °C for later use.

### 2.3. Protein Bradford Quantification

Total protein concentration was measured using the Bradford method [22]. Eleven Eppendorf (EP) tubes were separately labelled, and the protein standard solution BSA (1 mg mL^−1^) was accurately weighed into volumes: 0, 2, 4, 6, 8, 10, 12, 14, 16, 18, 20 μL. Corresponding to the EP tube, the test sample was taken as 1 μL. Each tube was then added with the corresponding double volume of deionized water and 180 μL working fluid (Table 1). The mixture was then vortexed for 20 s, mixed, centrifuged and reacted at 60 °C for 1 h. The absorbance was measured at 575 nm. A standard curve was prepared: y = 0.3927x − 0.0048, *R*^2^ = 0.9914; the quantitative results are shown in Table 2.

### 2.4. Digestion and Desalting

For each sample, 100 μg of protein was dissolved in 100 μL TEAB dissolution buffer and then diluted with 500 μL (50 mM) NH_4_HCO_3_. After the reduction of disulfides and alkylation, 2 μg trypsin was added and then incubated overnight at 37 °C for protein digestion. An equal volume of 0.1% FA was then added for acidizing. Peptides were purified on the Strata-X C18 pillar, which was first activated with methanol and then balanced by adding 1 mL 0.1% FA for three times, washed with 0.1% FA + 5% ACN twice, and eluted with 1 ml 0.1% FA + 80% ACN. Eluted peptides were then dried with a vacuum concentration meter. The dried peptide powder was re-dissolved with 20 μL (0.5 M) TEAB for peptide labelling.

### 2.5. iTRAQ Labeling and Fractionation

The samples were labelled with iTRAQ Reagent-8 plex Multiplex Kit (AB Sciex U.K. Limited, Shanghai, China) according to the manufacturer’s instructions. All of the labelled samples were mixed in equal amounts. The labelled samples were then fractionated by high-performance liquid chromatography (HPLC) system (Thermo DINOEX Ultimate 3000 BioRS, Waltham, MA, USA) using a Durashell C18 analytical column (5 μm, 100 Å, 4.6 × 250 mm). Finally, 12 fractions were collected.

### 2.6. Liquid Chromatography-Tandem Mass Spectrometry (LC-MS/MS) Analysis

Liquid chromatography-tandem mass spectrometry (LC- MS/MS) analysis was performed on an AB SCIEX nano-LC-MS/MS (Triple TOF 5600 plus) system. Samples were chromatographed using a 90 min gradient from 2–30% (buffer A; 0.1% (*v*/*v*) formic acid, 5% (*v*/*v*) acetonitrile: buffer B; 0.1% (*v*/*v*) formic acid, 95% (*v*/*v*) acetonitrile) after injecting into the AB SCIEX column system. MS1 spectra were collected in the range 350–1500 *m*/*z* for 250 ms. The 20 most intense precursors with charge state 2–5 were selected for fragmentation. MS2 spectra were collected in the range 50–2000 *m*/*z* for 100 ms; precursor ions were excluded from reselection for 15 s. The mass spectrometry results are shown in Table 3.

### 2.7. Protein Identification and Bioinformatics Analysis

The basic process of proteome identification based on mass spectrometry was adopted. The liquid chromatography-tandem mass spectrometry data was optimized by series and then compared with the database to score the protein for protein identification. MS/MS data for peptides were searched in the rice transcriptome database using Proteinpilot^TM^ V4.5. Unique peptide shows the number of unique peptide sequences for the proteome. Only proteins with at least one unique peptide and unused value more than 1.3 were considered for further analysis [23] and t-test was applied to it. When the difference was 1.5 times or more, (i.e., up-regulate ≥ 1.5 and down-regulate ≤ 0.67), it was regarded as a significantly different protein (*p*-value ≤ 0.05). The annotation function was used to perform gene function clustering (GO analysis) of differential proteins. The Kyoto encyclopedia of genes and genomes pathway database was used to analyze the metabolic pathways involved in differential proteins. The physical, chemical properties and distribution of the identified protein were graphically represented by Excel.

### 2.8. Real-Time PCR (qPCR) Verification

Some differential proteins were selected to perform the mRNA expression level verification in order to validate the iTRAQ results. Total RNA was extracted in accordance with the Trizol kit’s operating manual procedures and system. 1 μg of total RNA was taken and reversely transcribed into cDNA. The cDNA was served as a template. Real-time PCR internal reference gene (actin primers) were used in Q-PCR amplification to verify the mass of cDNA. The reaction conditions were (95 °C, 1 min, 1 cycle); (95 °C, 15 s, 60 °C, 40 cycles). The experiment was repeated thrice, and the relative expression was calculated by 2^−ΔΔCt^.

## 3. Result

### 3.1. The Mass Spectrometry Identification Result

**Figure 1:**
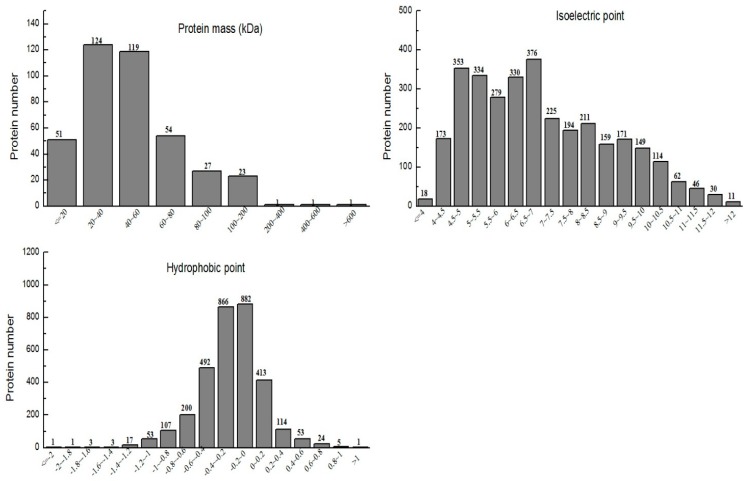
Protein mass distribution, isoelectric point distribution, and hydrophobic property analysis of identified proteins.

Mass spectrometry data were searched for in rice transcriptome databases via Proteinpilot^TM^ software (V4.5, Boston, MA, USA). A total spectrum of 3235 proteins with above 95% report confidence was identified. Out of these, 3161 proteins were quantified, which were further composed of 401 differential expressed proteins. Contingent significant differentially expressed protein analysis unveils a greater number of down-regulated (208) modified proteins in Se-enriched rice, and up-regulated (193) in non-Se-enriched rice. The molecular mass range of the protein was 8.2 kDa to 611.3 kDa, the isoelectric point range was 3.18 to 12.77, and the hydrophobicity range was −2.01 to 1.29, as shown in Figure 1. When the data of hydrophobicity was >0, the larger the value, the stronger the hydrophilicity. In comparison, when the data was <0, it was found that the smaller the value, the stronger was the hydrophobic effect.

### 3.2. Functional Annotation of Proteins

**Figure 2:**
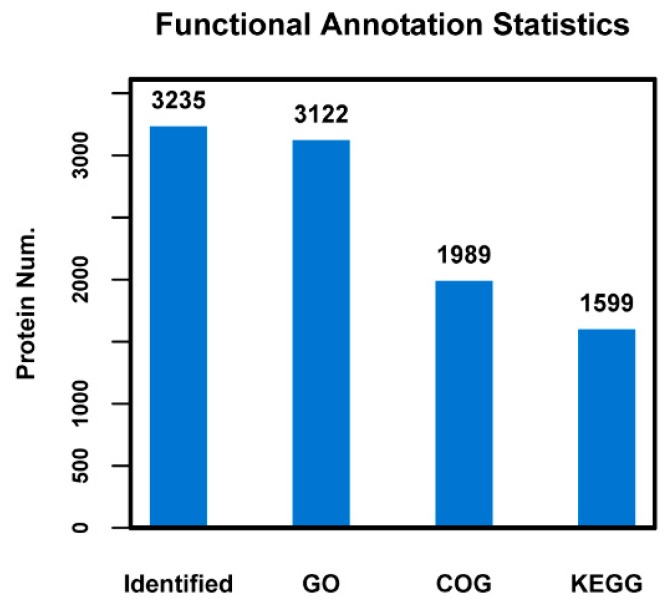
Statistical results of different functional annotations. The *x*-axis indicates identified or different annotation databases, and *y*-axis indicates protein number.

**Figure 3:**
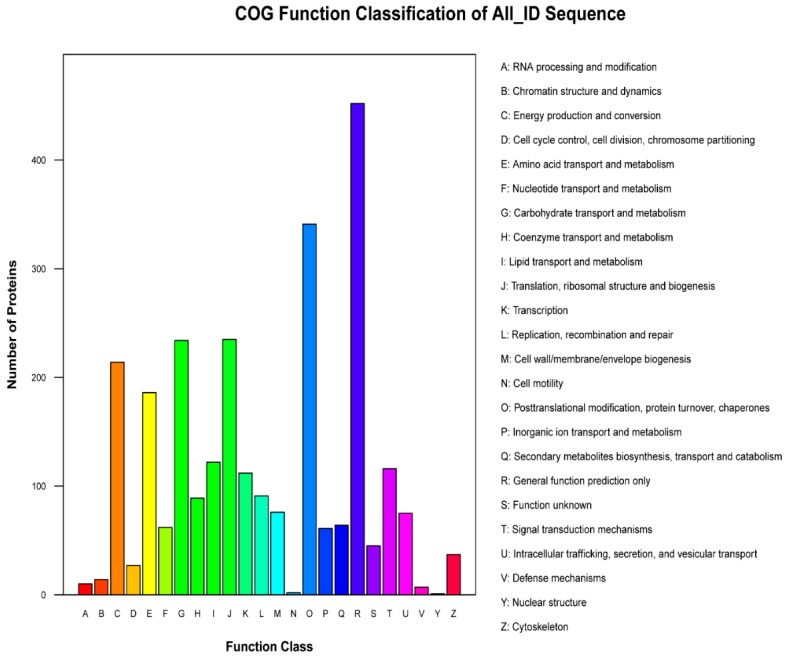
Cluster of Orthologous Groups (COG) function classification histogram of S727 and S3057 DEPs. The *x*-axis indicates different classification groups and the *y*-axis indicates the number of proteins in each COG class.

The GO, KEGG and COG annotation of the identified proteins were carried out to comprehensively reflect the biological function and significance of these proteins in various life activities. Functional annotation of all proteins obtained from Se-enriched and non-Se enriched rice revealed a sum of 3235 differential proteins. Of these, 3122 proteins were sub-categorized into 53 hierarchically-structured GO classifications (Figure 2). 1989 proteins were sub-categorized into 24 COG classifications. Class R (general function prediction only) was found to be significantly enriched and contains 452 proteins (Figure 2 and Figure 3). 1599 proteins identified for differential metabolic pathways by KEGG were sub-categorized into 116 classifications (Figure 2).

#### 3.2.1. Gene Ontology (GO) Annotation

**Figure 4:**
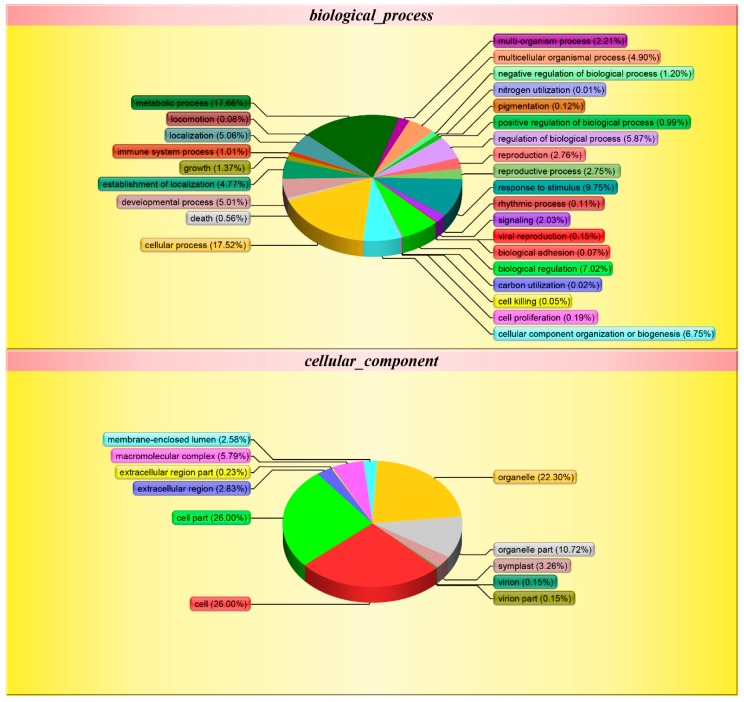

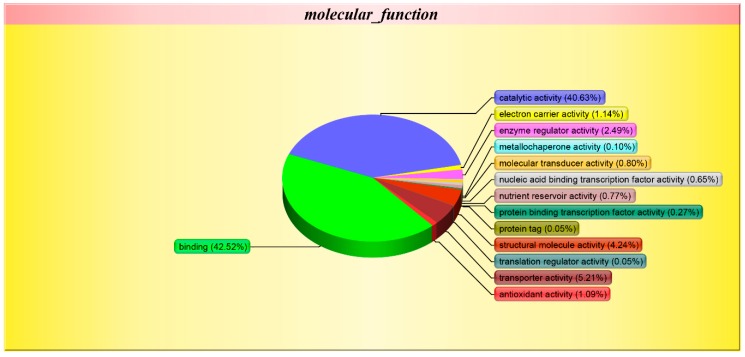
Gene ontology (GO) classification of the Differentially Expressed Proteins (DEPs) between S727 and S3057.

Gene ontology is a comprehensive approach, which indicates properties of genes and gene products in organisms. In order to get a detailed description, GO was further categorized into three components e.g. biological process, cellular component, and molecular function. Biological process-related GO terms between Se-enriched and non-Se-enriched rice disclose 401 differentially expressed proteins that mainly participate in 28 distinct functions. The biological process was found to be highly enriched in the ‘metabolic process’ (17.66%) and 17.52% for the ‘cellular process’ (Figure 4). Eleven ‘cellular component’ and 14 ‘molecular function’ related GO terms were found to be expressed the most. Cell and cell part were found to be enriched (26.00%) in the ‘cellular component’ category, while ionic binding (42.52%) and catalytic activity (40.63%) were most significantly expressed in ‘molecular function’. The differential GO analysis of Se-enriched and non-Se-enriched rice demonstrates a greater number of down-regulated genes than up-regulated ones.

#### 3.2.2. Cluster of Orthologous Groups (COG) Analysis

Cluster of Orthologous Groups of proteins is a database for the orthologous classification of proteins. We compared the identified differential proteins with the COG database to predict the possible functions of these proteins and then performed functional classification statistics on them (Figure 3). The top-5 most expressed COG class were: R, O, J, G and C. The number of expressed proteins in each class were 452, 341, 235, 234 and 214, respectively. The function inferred to each class was: general functional predictions (16.91%); protein conversion, translational modification, chaperone (12.76%); participation in translation, ribosome structure and biogenesis (8.8%); carbohydrate transport and metabolism (8.7%); and energy generation and conversion (8%). The result revealed that the differentially expressed proteins were involved in post-translational modifications, and carbohydrates and ribosomal transport. They also participated to some extent in energy production and amino acid transport. 

#### 3.2.3. Metabolic Pathway Annotation

The different proteins coordinate with each other in-vivo to express their biological behaviour. Thus, the pathway-based annotation broadens further understanding of their biological function. The KEGG is a main public pathway-related database (http://www.genome.jp/kegg/). The pathway analysis can determine important biochemical, and metabolic and signaling pathways regulated by proteins. The KEGG database results indicated that the differential proteins participated in 90 signaling pathways in total (Table 4). The top 10 metabolic pathways were starch and sucrose metabolic pathways (9.03%), glycolysis and gluconeogenesis pathway (9.03%), endoplasmic reticulum protein processing pathway (6.94%), ribosome metabolism (6.6%), photosynthetic biochar fixation pathway (5.21%), fructose and mannose metabolism (4.86%), galactose metabolism (4.17%), amino acids, nucleotides glucose metabolism (4.17%), purine metabolism (4.17%) and pyruvate metabolism (4.17%). The number of proteins expressed, and pathway ID is given in Table 4. 

#### 3.2.4. Functional Annotation of Differentially Expressed Proteins (DEPs)

**Figure 5:**
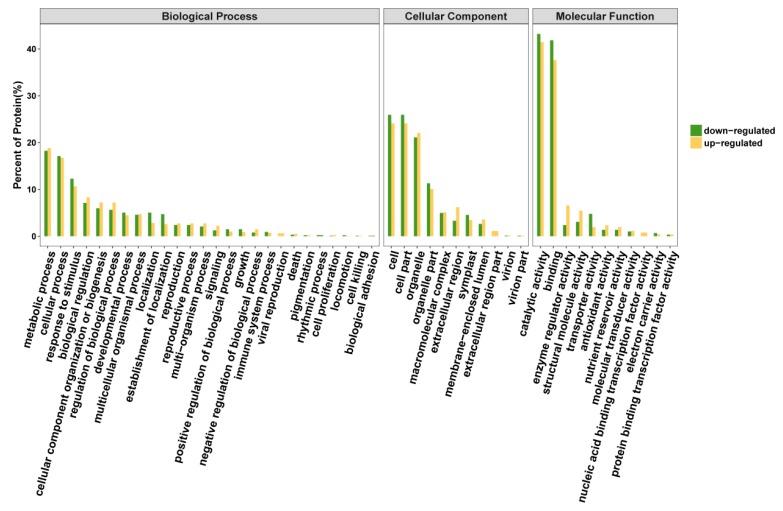
Gene ontology classification of DEPs between S727 and S3057.

The differences between up-regulated and down-regulated DEPs for some pathways were mesmerizing, as disclosed by GO (Figure 5). Some pathways like ‘extracellular region part’, ‘viral reproduction’ and ‘nucleic acid binding transcription factor activity’ were found to be expressed only in non-Se enriched rice. The Se responsive differential expressed proteins were mainly associated with diverse cellular functions that were related to cellular process, a main constituent of the cell and cell parts, ionic binding and catalytic activity (Figure 5).

**Figure 6:**
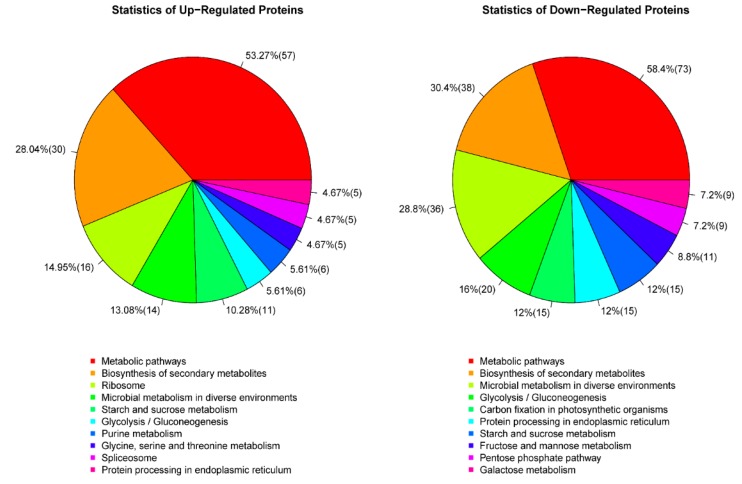
Statistics of Kyoto encyclopedia of genes and genomes (KEGG) pathways; DEPs in S727 (up-regulated proteins) and S3057 (down-regulated proteins).

The KEGG functional pathway statistical pie chart for the top 10 DEPs annotated to S727 and S3057 can be seen in Figure 6. It is observable from the results that expression of the top 10 functional pathways was different between Se-enriched and non-Se-enriched rice. It is evident from the GO, KEGG annotation analysis that the metabolic pathway was the most enriched pathway present in 401 differential proteins. Although the most common pathway annotated in both rice groups was the metabolic pathway, the trend of the concurrence of this pathway seems to be most in Se-enriched rice (73%). There were six same functions among the top 10 annotated pathways but the contingency of these pathways in both rice types were different, i.e., metabolic pathways; 73%:57%, biosynthesis of secondary metabolites; 38%:30%, microbial metabolism in diverse environments; 36%:14%, glycolysis/gluconeogenesis; 20%:6%, protein processing in endoplasmic reticulum; 15%:5%, starch and sucrose metabolism; 15%:11%, in Se-enriched and non-Se-enriched rice, respectively. The expression of annotated DEPs was more in the rice group responsive for Se. Thus, the Se-enriched rice seems to have better molecular functions and regulatory effects then non-Se-enriched rice. Se also seems to be an integral part of cellular components. 

#### 3.2.5. Screening for Protein Information

**Figure 7:**
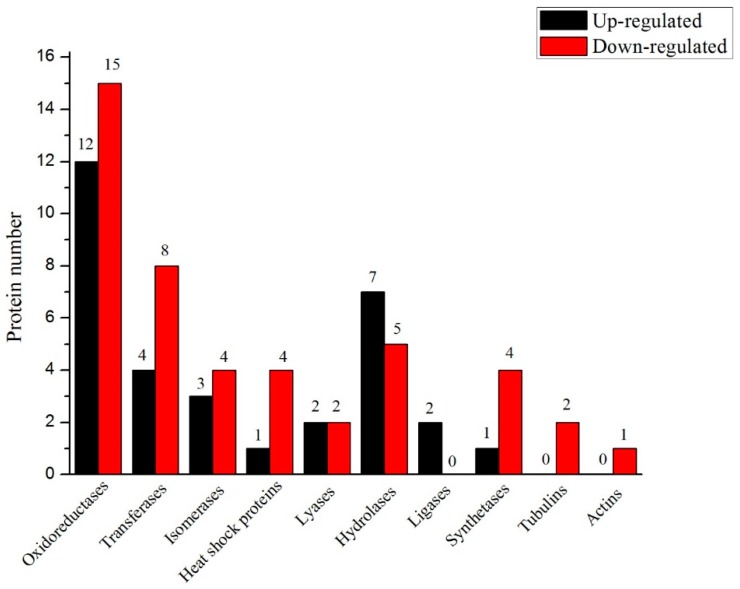
The ten categories of significantly differentially expressed proteins in S727 and S3057.

Finally, 77 targeted differential proteins were screened-out in accordance with the expression level, molecular function, and metabolic pathways (Figure 7). These proteins were further categorized according to their function as oxidoreductases, transferases, isomerases, heat shock proteins, lyases, hydrolases, ligases, synthetases, tubulins and actins. The number of proteins present in each class was: 27, 12, 7, 5, 4, 12, 2, 5, 2 and 1, respectively. The comparison of each class indicated that the number of proteins expressed in Se-enriched rice was more for most of the classes. The protein number for hydrolases and ligases were more in the non-Se-enriched rice. The proteins expressed for lyases function were the same in both rice types. 

#### 3.2.6. qPCR Verification of Differential Genes

**Figure 8:**
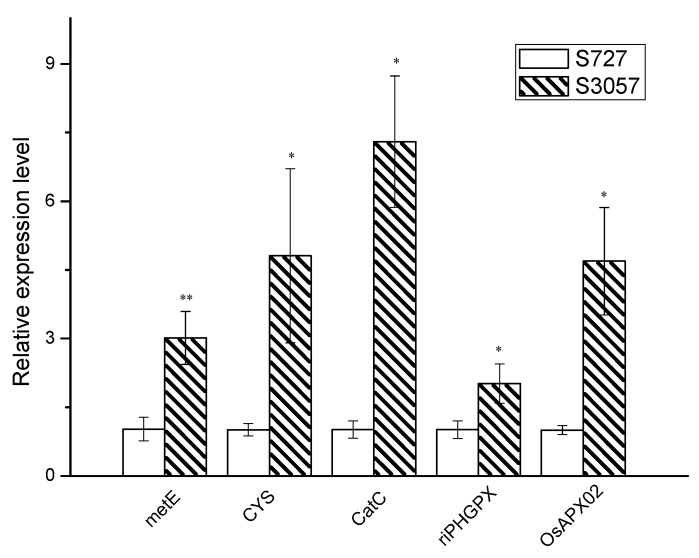
Relative abundance of selected DEPs revealed by realtime (qPCR) analysis.

The genome in an organism is the storage of genetic information. mRNA is the prerequisite of gene expression, and the protein level is the executive of the gene function. In order to verify whether the changes at the gene level are consistent with the protein level, qPCR verification was employed for the selected proteins. OsAPx02, CatC, riPHGPX, CYS and metE proteins were selected for qPCR verification (Figure 8). The results showed that the expression levels of *riPHGPX* and *metE* genes were consistent with the protein levels. In addition, the *OsAPx02*, *CatC* and *CYS* genes, which were down-regulated by S727 protein, disclosed an alternative insight at the mRNA level relative to S3057. Many studies have reported unrelated or negative correlations between proteomics and transcriptomes. The main reason behind it is probably the post-translational modification (phosphorylation, glycosylation, etc.) of proteins, affecting protein secretion and degradation [24]. Post-regulatory effects greatly influence the expression of different gene levels, while certain inconsistency in them leads to abnormal expression. The stability of mRNA after genetic transcription could be related to specific nucleotides and corresponding binding proteins [25].

## 4. Discussion

Protein is both the specific executive of life activities and the embodiment of the final life function. Its integrity cannot be denied, and the countless indispensable functions performed by proteins are still the debate of the century. However, to fully exploit natural genetic information, efforts should be made to develop and utilize novel tools. Proteomics is an emerging novel tool to study proteins. An integrated proteomic approach (iTRAQ), with the combined use of high throughput mass spectrometry (LC-ESI-MS/MS), was employed. The basic essential functions performed by proteomics are: (i) Proteomics can study proteins with specific physiological functions on a large scale, and then clarify the information about the whole protein. (ii) It can obtain the qualitative and quantitative information of key proteins to effectively study the function and interaction of proteins, protein expression, and post-translational modification, etc. (iii) The presence of differentially expressed proteins may lead to differences in the accumulation of secondary metabolites. Therefore, protein expressional change identification is critical and has deep regularity effects. It will, therefore, be helpful in identifying the mechanism of Se-enrichment in rice. 

Results of the comparative annotation analysis of Se-enriched and non-Se-enriched rice demonstrated significant differences at the proteome expression level. A series of bioinformatic analysis pointed out the presence of 401 differential proteins. Of these, 77 targeted differential proteins were divided into 10 groups: oxidoreductase, transferase, isomerase, heat shock protein, lyase, hydrolase, ligase, synthetase, tubulin, and actin, based on their functions. The functional distribution is further discussed in Table 5. Oxidoreductase proteins mainly participate in the anti-stress and bioregulation synthesis process. Among the 27 identified oxidoreductases, there were 12 up-regulated and 15 down-regulated proteins in non-Se-enriched rice relative to the natural Se-enriched rice. OsAPx02, CatC and riPHGPX proteins were identified to have high expression. The first two were up-regulated proteins, and the latter was down-regulated protein. The OsAPx02 belongs to the APX (ascorbate peroxidase) gene family, and it is one of the important members of the ROS scavenging system. It participates in many reactive oxygen metabolism processes in cells and plays an active role in maintaining normal cell metabolism. The studies showed that the OsAPx02 gene can enhance the growth and development of rice in drought, salt, and low-temperature resistant environments [18,26]. CatC is an important antioxidant and key enzyme in the defence system established by plants during growth. CatC can inhibit the excessive growth of H_2_O_2_, and is thus an important enzyme in the plant that controls H_2_O_2_ levels and the redox balance of plant cells [27]. CatC plays an important role in stress resistance, and it can improve the defence ability of rice [28]. The riPHGPX belongs to the GSH-Px (glutathione peroxidase) gene family, and it plays an important role by regulating and catalyzing the redox state in cells [29]. The number of down-regulated proteins was higher than that of the up-regulated proteins in the oxidoreductase group. Thus, the oxidoreductase’s anti-stress, anti-oxidation and reactive oxygen catabolism in natural Se-enriched rice were better than that in non-Se enriched rice.

Transferases proteins are mainly found to be a participant in metabolic processes i.e., carbohydrate and intracellular amino acid. Among the 12 transferases, there were four up-regulated in non-Se-enriched rice and eight down-regulated proteins in natural Se-enriched rice. CYS and metE proteins were found to have high expression. The former was the up-regulated protein, and the latter was the down-regulated protein. CYS can catalyze plants to synthesize cysteine [30]. The metE participated in the synthesis of methionine and linked important functions such as protein synthesis, methyl transfer, polyamine and ethylene synthesis, to cell metabolism [31], as metE protein expression was found more in Se-enriched rice. Therefore, the carbohydrate and intracellular amino acid metabolism ability of transferase in natural Se-enriched rice were higher. 

There were seven targeted differential proteins in the isomerase class, including three up-regulated and four down-regulated proteins. Isomerases proteins are known for their functions viz., stress resistance, plant carbohydrate and nitrogen-containing compound metabolism. OsI_05445 belonged to the protein disulfide isomerase (PDI) gene family and found high expression in Se-enriched rice. It mainly participated in the repair of damaged proteins under adverse stress to promote the synthesis of nascent peptides [32,33], which indicated that the resistance ability of natural Se-enriched rice was better under stress conditions than that of the non-Se-enriched rice. Among the five targeted differential heat shock proteins (HSP), one was up-regulated, and four differential proteins were down-regulated. Biological processes of anti-stress and protein folding are mainly controlled by HSP. Os01g0180800 and OJ1540_H01.1 were found to have the highest expression. Os01g0180800 protein belongs to the Hsp70 gene, which is the most important and conserved HSP family. Hsp70 represents constitutive expression in-vivo. Under heat shock conditions, this protein found significant expression and control sorting of the nascent peptide chain, maturation as well as the transport of secreted proteins to the extracellular organelles [34,35]. OJ1540_H01.1 protein belongs to Hsp90, which is also a highly conserved heat shock protein. It functions in the correct regulation of various proteins in plant cells, while also ensuring cell stability under adverse stress conditions [36]. Both the studied HSPs found with maximum expression were down-regulated proteins. Hence, the anti-stress participation ability of natural Se-enriched rice was better than that of non-Se-enriched rice.

Lyases proteins are known for their functions in the biosynthesis process. In total, four lyases proteins were found with attribution of two up-regulated and two down-regulated proteins. Glutamate decarboxylase (GAD) was a down-regulated protein and was found with high expression. Glutamate decarboxylase is a key enzyme for the synthesis of γ-aminobutyric acid (GABA), and it had many biological functions, such as promoting brain activity, calming the nerves, regulating hormone secretion, lowering blood pressure, treating epilepsy, enhancing memory, and improving menopausal syndrome. As compared with non-transgene, transgenic plants have a higher GAD activity and γ-aminobutyric acid content, which indicates that GABA accumulation can be achieved by enhancing GAD expression via genetic engineering [37]. Therefore, lyases’ role in Se-enriched rice was evident. Hydrolases are important for cell tissue regulation and biosynthesis processes. Out of 12, 7 up-regulated and 5 down-regulated proteins were found. PUL (limit dextrinase) and OsI_26372 were up-regulated proteins and found with most expression. PUL belongs to the starch debranching enzyme (DBE) in the starch synthesis pathway and is expressed at a high level in both the middle and late stages of seed development [38]. These two ligases contribute to the metabolism of small molecules, and both were up-regulated proteins. Therefore, the role of non-Se-enriched rice in starch synthesis pathway was slightly better than that of the natural Se-enriched rice.

One up-regulated synthetases protein was found out of five, while the rest were down-regulated proteins. Anti-stress and glucose metabolism processes were controlled by them. The annotation analysis revealed a down-regulated protein, sucrose synthase (SUS), with the highest proteinic expression. Sucrose synthase is the key enzyme for plant sucrose metabolism [39] and controls nitrogen-fixing biosynthesis and abiotic stress reaction processes [40,41]. It is designated that the glucose metabolism of synthetase in natural Se-enriched rice was better than that in non-Se-enriched rice. In addition, there were two tubulins and one actin, which was all down-regulated proteins [41,42] that played key roles in maintaining cell shape, movement, and signaling. Hence, signaling pathways and cell movement features of Se-enriched rice were better than non-Se-enriched rice. This study provided the basic data for secondary metabolic differences among different rice genotypes and uncovered important information at the proteinic level for further studies on Se-enriched food. 

## 5. Conclusions

A quantitative proteomics study was conducted on rice, based on iTRAQ technology, to find out the difference between natural Se-enriched and non-Se-enriched rice at proteome differential expression levels. Bioinformatic analysis on differential proteins pointed out anti-stress, anti-oxidation, reactive oxygen metabolism, carbohydrate and amino acid metabolism of natural Se-enriched rice was better than non-Se-enriched rice. Howbeit, the starch synthesis pathway was evidently more in non-Se enriched rice. It can be inferred from GO, COG and KEGG annotations that CYS and metE probably were the two key proteins that caused amino acid differences between these two types of rice, and OsAPx02, CatC, riPHGPX, HSP70 and HSP90 perhaps were the key enzymes regulating the antioxidant and anti-stress effects in these two types of rice. This study provides interesting insights on proteome analysis and proteinic differential expression in Se-enriched and non-Se-enriched rice. In nature, Se is the key contributor to many biological and metabolic processes and has scavenging effects. However, the present investigation nurtures our understanding of the functioning of this trace element and uncovers the protein mechanism underlying Se.

## Figures and Tables

**Table 1:** Bradford quantitative standard curve production process.

Pipe Number	0	1	2	3	4	5	6	7	8	9	10
Double distilled water/μL	0	2	4	6	8	10	12	14	16	18	20
BSA/μL	20	18	16	14	12	10	8	6	4	2	0
Working fluid/μL	180	180	180	180	180	180	180	180	180	180	180
Final concentration (μg/μL)	0.20	0.18	0.16	0.14	0.12	0.1	0.08	0.06	0.04	0.02	0

**Table 2:** Sample Bradford quantitative results.

Sample number	S727	S3057
Sample absorbance	0.264	0.268
Determination of concentration (μg/μL)	0.099	0.100
Dilution factor	100	100
Actual sample concentration (μg/μL)	9.9	10.0

**Table 3:** Liquid chromatography-tandem mass spectrometry (LC-MS/MS) results.

Time (min)	0	65	70	80	85	90
% A	95	70	50	20	20	95
% B	5	30	50	80	80	5

**Table 4:** Pathway enrichment analysis of differentially expressed proteins.

Pathway	Number of Proteins	Pathway ID
Starch and sucrose metabolism	26	KO00500
Glycolysis/gluconeogenesis	26	KO00010
Protein processing in endoplasmic reticulum	20	KO04141
Ribosome	19	KO03010
Carbon fixation in photosynthetic organisms	15	KO00710
Fructose and mannose metabolism	14	KO00051
Galactose metabolism	12	KO00052
Amino and nucleotide sugar metabolism	12	KO00520
Purine metabolism	12	KO00230
Pyruvate metabolism	12	KO00620

**Table 5:** Significantly differentially expressed proteins in S727 and S3057.

Accession Number	Protein Name	Gene Name	Protein Mass	% Cov	Peptides	Peak Area Ration of MS/MS (S727:S3057)
**Oxidoreductases**						
tr|Q8W5H7|Q8W5H7_ORYSJ	ascorbate peroxidase	*OsAPx02*	34.0	34.88	79	2.253
tr|Q9ZRI9|Q9ZRI9_ORYSJ	Catalase	*CatC*	112.7	19.92	76	1.031
tr|Q9FEV2|Q9FEV2_ORYSA	Glutathione peroxidase	*riPHGPX*	91.2	9.47	61	0.788
tr|B8AF09|B8AF09_ORYSI	Glyceraldehyde-3-phosphate dehydrogenase	*OsI_07948*	38.6	75.28	38	0.280
tr|I3QD45|I3QD45_ORYSJ	Glyceraldehyde-3-phosphate dehydrogenase	GAPDH	36.4	73.29	27	0.374
tr|Q84VE1|Q84VE1_ORYSJ	Adenosylhomocysteinase	*OsJ_33804*	53.2	56.08	26	0.557
tr|E3WF37|E3WF37_ORYSJ	Lysine ketoglutarate reductase/saccharopine dehydrogenase	*OsLKR/SDH*	116.7	32.33	26	2.698
sp|P0C5C8|REHYA_ORYSI	1-Cys peroxiredoxin A	*OsI_27030*	24.1	90.45	24	0.065
tr|Q9FRX7|Q9FRX7_ORYSJ	Aldehyde dehydrogenase ALDH2b	*Aldh2b*	59.3	55.37	24	0.604
tr|A0A0B4U151|A0A0B4U151_ORYSA	6-phosphogluconate dehydrogenase	*LOC_Os06g02144.1*	52.7	58.33	20	0.431
tr|B8AIJ7|B8AIJ7_ORYSI	Aldehyde dehydrogenase	*OsI_06045*	56.1	38.14	18	0.351
tr|Q6H703|Q6H703_ORYSJ	Glyceraldehyde-3-phosphate dehydrogenase	*Os02g0171100*	43.3	47.45	17	1.965
tr|Q10C90|Q10C90_ORYSJ	Aldehyde oxidase	*LOC_Os03g57690*	145.1	16.24	16	0.482
tr|Q43803|Q43803_ORYSA	Superoxide dismutase	*rmsod2*	25.0	79.22	14	0.272
sp|Q33E23|DHE2_ORYSJ	Glutamate dehydrogenase 2, mitochondrial	*GDH2*	44.6	50.61	14	1.966
tr|B9V0M0|B9V0M0_ORYSI	Alcohol dehydrogenase family-2	*OSI9Ba083O10_092B13-4*	41.2	59.63	14	0.643
sp|Q84LK3|BADH2_ORYSJ	Betaine aldehyde dehydrogenase 2	*BADH2*	54.7	40.56	12	2.098
sp|Q6Z5N4|ODPA1_ORYSJ	Pyruvate dehydrogenase E1 component subunit alpha-1	*Os02g0739600*	42.7	44.36	12	1.960
tr|Q8SAZ7|Q8SAZ7_ORYSJ	V-type proton ATPase subunit	*OSJNBa0029P16.14*	88.8	19.28	11	0.491
sp|Q7XI14|D2HDH_ORYSJ	Probable D-2-hydroxyglutarate dehydrogenase	*D2HGDH*	61.1	26.48	11	0.515
tr|Q6ZI55|Q6ZI55_ORYSJ	NAD-dependent isocitrate dehydrogenase c	*OsIDHc*	40.6	40.74	10	1.869
tr|Q10BJ7|Q10BJ7_ORYSJ	Methylenetetrahydrofolate reductase	*LOC_Os03g60090*	66.4	21.89	10	0.569
tr|Q6ZKB1|Q6ZKB1_ORYSJ	Dihydrolipoamide acetyltransferase component of pyruvate dehydrogenase complex	*Os08g0431300*	48.8	22.53	8	1.821
tr|Q94DM0|Q94DM0_ORYSJ	Peroxidase	*prx23*	37.9	34.64	7	0.190
tr|A3BVS6|A3BVS6_ORYSJ	Superoxide	*OsJ_28291*	20.5	70.94	7	2.577
tr|Q9SXM1|Q9SXM1_ORYSA	Cysteine endopeptidase	*Rep1*	40.7	22.91	5	5.101
tr|B8ARK2|B8ARK2_ORYSI	Amine oxidase	*OsI_15134*	24.1	7.60	3	1.955
**Transferases**						
sp|Q9XEA8|CYSK2_ORYSJ	Cysteine synthase	*CYS*	145.1	61.23	65	1.025
tr|Q10S41|Q10S41_ORYSJ	Methyltransferase	*metE*	229.9	62.76	41	0.956
tr|H2KWU8|H2KWU8_ORYSJ	5-methyltetrahydropteroyltriglutamate-homocysteine methyltransferase	*LOC_Os12g42884*	84.6	35.51	25	0.140
sp|Q0J8G4|SCRK2_ORYSJ	Fructokinase-2	*FRK2*	35.5	52.08	15	3.632
tr|Q6WSC2|Q6WSC2_ORYSI	Glutathione S-transferase	*gstu4*	25.3	41.20	13	0.268
tr|Q01IL1|Q01IL1_ORYSA	ATP-dependent 6-phosphofructokinase	*OSIGBa0150F01.9*	51.3	37.74	13	0.595
sp|A2WXV8|SCRK1_ORYSI	Fructokinase-1	*FRK1*	34.7	53.56	12	1.874
tr|Q10BJ7|Q10BJ7_ORYSJ	Methylenetetrahydrofolate reductase	*LOC_Os03g60090*	66.4	21.89	10	0.569
tr|Q65X97|Q65X97_ORYSJ	ATP-dependent 6-phosphofructokinase	*PFK*	62.6	17.11	8	0.418
tr|B8ANV9|B8ANV9_ORYSI	1,2-dihydroxy-3-keto-5-methylthiopentene dioxygenase	*OsI_10122*	23.5	41.92	7	1.741
tr|A2YPU1|A2YPU1_ORYSI	Glycosyltransferase	*OsI_27293*	51.0	24.05	7	0.499
tr|Q8W2T8|Q8W2T8_ORYSJ	Glutathione S-transferase, N-terminal domain containing protein	*Os10g0365200*	26.1	15.73	4	0.523
**Isomerases**						
tr|B8AGU2|B8AGU2_ORYSI	Protein disulfide-isomerase	*OsI_05445*	62.3	60.39	38	4.079
tr|B8BCM8|B8BCM8_ORYSI	Glucose-6-phosphate isomerase	*OsI_31689*	68.4	42.24	19	0.536
tr|Q8H3Q7|Q8H3Q7_ORYSJ	Xylose isomerase	*P0625E02.119*	53.5	51.15	18	0.339
tr|Q7F1F2|Q7F1F2_ORYSJ	Peptidylprolyl isomerase	*OJ1191_A10.119*	64.1	31.21	14	0.574
sp|Q84T92|CFI_ORYSJ	Chalcone--flavonone isomerase	*CHI*	23.9	60.09	10	0.508
tr|B8ACM2|B8ACM2_ORYSI	Peptidyl-prolyl cis-trans isomerase	*OsI_01440*	25.4	27.31	5	3.737
tr|B9FFI3|B9FFI3_ORYSJ	Aldose 1-epimerase	*OsJ_15041*	40.5	27.89	4	4.381
**Heat shock protein**						
tr|Q7X9A7|Q7X9A7_ORYSJ	60 kDa chaperonin alpha subunit	*LOC_Os03g64210*	61.4	73.63	39	0.468
tr|Q8SB39|Q8SB39_ORYSA	Heat shock protein 90	*OJ1540_H01.1*	93.0	45.69	36	0.344
tr|Q9AQZ5|Q9AQZ5_ORYSJ	70 kDa heat shock protein	*Os01g0180800*	93.1	41.78	32	0.372
tr|Q8H903|Q8H903_ORYSJ	60 kDa chaperonin	*Os10g0462900*	60.8	62.02	27	0.656
tr|Q8H3I7|Q8H3I7_ORYSJ	10 kDa chaperonin	*P0524G08.116*	10.6	58.16	7	1.998
**Lyases**						
tr|A2YW09|A2YW09_ORYSI	Glutamate decarboxylase	*GAD*	58.2	30.93	11	0.275
tr|B9FNQ3|B9FNQ3_ORYSJ	Lactoylglutathione lyase	*OsJ_17937*	26.3	59.92	11	4.188
tr|Q5W6C5|Q5W6C5_ORYSJ	Carboxypeptidase	*Os05g0268500*	53.8	24.02	8	3.086
tr|Q67IU5|Q67IU5_ORYSJ	Ribulose bisphosphate carboxylase small chain	*Os02g0152400*	20.6	15.30	3	0.653
**Hydrolases**						
tr|D0TZH3|D0TZH3_ORYSI	Pullulanase	*PUL*	102.5	76.25	79	51.110
tr|A2X5K0|A2X5K0_ORYSI	Starch branching enzyme 3	*SBE3*	92.8	57.70	59	0.661
tr|A2X2G9|A2X2G9_ORYSI	Aminopeptidase	*OsI_06386*	98.4	36.33	25	0.315
tr|B8BAI7|B8BAI7_ORYSI	Aminopeptidase	*OsI_29144*	97.9	35.89	24	0.636
tr|Q0J528|Q0J528_ORYSJ	Alpha-amylase	*Os08g0473600*	48.7	43.71	14	2.819
sp|Q0INM3|BGA15_ORYSJ	Beta-galactosidase 15	*Os12g0429200*	101.0	23.94	15	0.653
tr|A0MLU9|A0MLU9_ORYSI	Alpha-amylase /trypsin inhibitor	*OsI_25388*	15.8	69.59	16	3.576
tr|A2YMB7|A2YMB7_ORYSI	Beta-amylase	*OsI_26372*	55.2	31.35	10	25.325
tr|Q8H484|Q8H484_ORYSJ	Beta-amylase	*P0450A04.127*	56.8	30.02	9	10.968
tr|Q5W6C5|Q5W6C5_ORYSJ	Carboxypeptidase	*Os05g0268500*	53.8	24.02	8	3.086
tr|D0TZH0|D0TZH0_ORYSI	Pullulanase	*PUL*	102.5	72.98	78	0.026
tr|B8BCL1|B8BCL1_ORYSI	Alpha-amylase	*OsI_31647*	48.9	14.55	5	2.160
**Ligases**						
tr|B8BFZ6|B8BFZ6_ORYSI	Alanine--tRNA ligase	*OsI_32912*	104.8	30.75	25	1.732
tr|Q6Z050|Q6Z050_ORYSJ	Polyadenylate-binding protein	*P0451H06.101*	71.6	25.91	11	2.008
**Synthetase**						
tr|A2YNQ2|A2YNQ2_ORYSI	Sucrose synthase	*SUS*	93.1	61.03	61	0.413
sp|B9EXM2|CARB_ORYSJ	Carbamoyl-phosphate synthase large chain	*CARB*	127.8	27.39	22	0.606
sp|A2XD35|PURA_ORYSI	Adenylosuccinate synthetase	*PURA*	52.0	49.08	20	3.110
tr|Q10RX2|Q10RX2_ORYSJ	Cysteinyl-tRNA synthetase	*LOC_Os03g04960*	60.9	45.64	18	0.512
sp|Q6L4S0|DDB1_ORYSJ	DNA damage-binding protein 1	*DBB1*	121.9	15.23	10	0.511
**Tubulin**						
tr|Q0PVB0|Q0PVB0_ORYSJ	Tubulin alpha chain	*TubA*	49.7	55.11	19	0.357
tr|Q9FVI3|Q9FVI3_ORYSA	Tubulin alpha chain	*TubA1*	49.8	57.87	17	0.384
**Actin**						
tr|Q75HX0|Q75HX0_ORYSJ	Actin	*Os05g0438800*	41.6	65.43	19	0.411
